# Optimal Cleavage and Oxidative Folding of α-Conotoxin TxIB as a Therapeutic Candidate Peptide

**DOI:** 10.3390/md11093537

**Published:** 2013-09-17

**Authors:** Xiaosa Wu, Yong Wu, Furong Zhu, Qiuyuan Yang, Qianqian Wu, Dongting Zhangsun, Sulan Luo

**Affiliations:** Key Laboratory of Tropical Biological Resources, Ministry of Education, Key Lab for Marine Drug of Haikou, Hainan University, Haikou, Hainan 570228, China; E-Mails: 7776820@163.com (X.W.); wys211@163.com (Y.W.); 704242191@qq.com (F.Z.); yang.qiuyuan@hotmail.com (Q.Y.); 541214749@qq.com (Q.W.)

**Keywords:** α-conotoxin TxIB, oxidative folding, optimization, therapeutic peptides

## Abstract

Alpha6beta2 nicotinic acetylcholine receptors (nAChRs) are potential therapeutic targets for the treatment of several neuropsychiatric diseases, including addiction and Parkinson’s disease. Alpha-conotoxin (α-CTx) TxIB is a uniquely selective ligand, which blocks α6/α3β2β3 nAChRs only, but does not block the other subtypes. Therefore, α-CTx TxIB is a valuable therapeutic candidate peptide. Synthesizing enough α-CTx TxIB with high yield production is required for conducting wide-range testing of its potential medicinal applications. The current study optimized the cleavage of synthesized α-CTx TxIB resin-bounded peptide and folding of the cleaved linear peptide. Key parameters influencing cleavage and oxidative folding of α-CTx TxIB were examined, such as buffer, redox agents, pH, salt, co-solvent and temperature. Twelve conditions were used for cleavage optimization. Fifty-four kinds of one-step oxidative solution were used to assess their effects on each α-CTx TxIB isomers’ yield. The result indicated that co-solvent choices were particularly important. Completely oxidative folding of globular isomer was achieved when the NH_4_HCO_3_ or Tris-HCl folding buffer at 4 °C contained 40% of co-solvent DMSO, and GSH:GSSG (2:1) or GSH only with pH 8~8.7.

## 1. Introduction

Neuronal nicotinic acetylcholine receptors (Alpha6beta2 nicotinic acetylcholine receptors (nAChRs)) play a crucial role in central and peripheral nervous system and development of many neuropsychiatric diseases, such as Parkinson’s disease, nicotine addiction, *etc*. [1]. These pentameric receptors are members of the Cys-loop family of ligand-gated ion channels, composed of a combination of α and β subunits [2]. Depending on specific receptor composition, nAChR receptor subtypes have distinct pharmacological properties and physiological functions. For example, compared to other nAChR receptors, α4β2 nAChR has the highest affinity for nicotine [3]; α6β2*-nAChRs are involved in the pathophysiology of Parkinson’s disease, as well as addiction [4]. The α9α10 nAChR is an important target for the development of analgesics and cancer chemotherapeutics [5].

**Figure 1:**
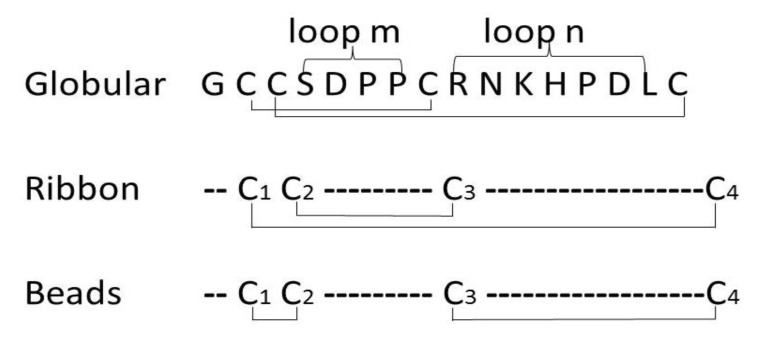
Schematic representation of globular, ribbon and bead disulfide connectivities of α4/7-conotoxin TxIB.

Therapeutic targeting of nAChRs shows great promise for treatment of nervous system diseases. Investigators around the world have identified conotoxins (CTxs) from different species of marine cone snails (*Conus*) as a possible source for developing such medications [6,7]. α-conotoxins (α-CTxs) are 12–20 amino acid disulfide-constrained peptides that target diverse nAChR subtypes [8,9,10,11,12]. Prior research has identified α-CTxs as important research tools, both for studying diverse pharmacological disorders and for developing new therapeutics for treating addiction, neuropathic pain and other neuropathological conditions [13]. All α-CTxs share a conserved cysteine pattern, CC-C-C. Amino acid residues between Cys 2 and 3 are identified as *m*-loop; residues between Cys3 and Cys4 are identified as *n*-loop. The number of residues in each loop is used for α-CTx classification. Disulfide bonds of native α-CTx isomers are usually connected in a (CysI–III, CysII–IV) “globular” arrangement. In contrast, an alternative “ribbon” arrangement has Cys1–4, Cys2–3 bonding, while a “beads” arrangement has CysI–II, CysIII–IV bonding (Figure 1). α-CTx activity against specific targets is highly dependent on cysteine bond arrangement [14].

Our laboratory has recently identified α-CTx TxIB as a highly selective blocker of α6/α3β2β3 nAChRs, with little or no activity against other receptor subtypes [15]. α6β2* nAChRs are potential targets for the treatment of several neuropsychiatric diseases, including addiction and Parkinson’s disease. Therefore, α-CTx TxIB is a valuable therapeutic candidate peptide. TxIB is classified as α4/7-conotoxin, since it has four residues in the *m*-loop and seven residues in the *n*-loop (Figure 1). High-yield production of α-CTx TxIB is an essential first step for conducting wide-range testing of its potential medicinal applications. Therefore, the current study focused on developing optimal cleavage conditions and efficient oxidation folding methods for TxIB production.

One of the most important steps in peptide synthesis is cleavage and deprotection. Unless suitable reagents and reaction conditions are chosen, newly synthesized peptide would be irreversibly modified or damaged. Most cleavage and deprotection techniques are trifluoroacetic acid (TFA)-based. However, TFA cleavage also generates several highly reactive species, often resulting in covalent modification of susceptible residues [16]. All of the TFA concentration, types of scavengers used and reaction time influence cleavage reaction efficiency [17,18]. Therefore, appropriate scavengers and reaction conditions must be chosen for optimal cleavage yield.

Oxidative folding of Cys-rich peptides in endoplasmic reticulum (ER) milieu involves numerous interactions, which cannot be reproduced *in vitro*. Generally, it is difficult to obtain sufficient quantities of appropriately folded isomers of small, cys-rich peptides * in vitro*, which preclude one from efficient production of synthetic bioactive peptides [19,20,21]. Prior studies primarily relied on two approaches for oxidative folding of conotoxins: two-step regioselective oxidation and one-step random oxidation. One-step oxidation, while in principle having lower selectivity compared to regioselective oxidation, involves fewer purification steps and can be experimentally optimized for better yield. Although such optimization requires extensive testing, overall yield might be significantly greater compared to two-step regioselective oxidation.

In this study, we investigated optimal conditions for cleavage and one-step folding of TxIB. To improve final yields of TxIB globular isomer and to determine key parameters that influence disulfide formation in the TxIB folding process, we systematically examined 12 cleavage conditions, which include four different cleavage cocktails and three different cleavage times. We also tested 54 kinds of oxidative folding solutions involving different buffers, redox reagents, pH, temperature, co-solvents and reaction times.

## 2. Results and Discussion

### 2.1. Results

#### 2.1.1. Fmoc Synthesis and Cleavage of α-CTx TxIB Resin-Bounded Peptide

**Figure 2:**
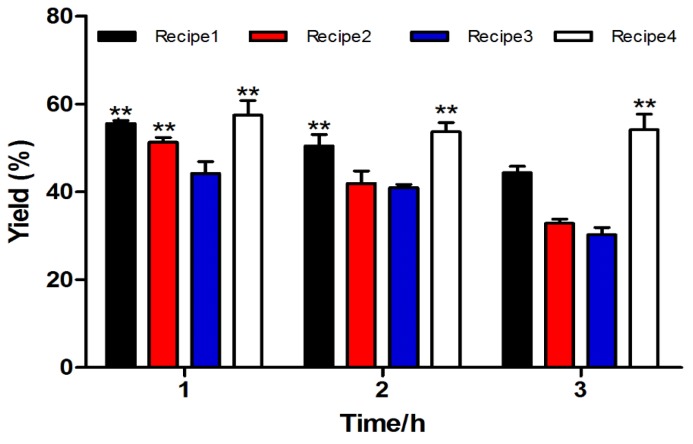
Yield of alpha-conotoxin (α-CTx) TxIB resin-bounded peptide undergoing different cleavage conditions. The two-way ANOVA/Tukey test and three replicates were used for analysis, and data were expressed as the mean ± SEM (** *p* < 0.01); the values were compared to Recipe 3.

The success of solid phase peptide synthesis is affected by disadvantageous side reactions that occur during TFA peptide-resin cleavage and side-chain deprotection. The bulk of these side reactions modifies susceptible residues with TFA-liberated side-chain protecting groups and linkers. Optimizing final TFA concentration, the types of scavengers used and reaction time helps to suppress undesired side reactions and improve yields. We chose four different cleavage cocktails and three different reaction time scales to discover the best cleavage solution for the resin-bounded peptide of α-CTx TxIB by one-step oxidation synthesis (Table 1). Cleavage efficiency and yield were significantly influenced by the reaction conditions (Figure 2). Cleaved crude linear peptide was purified by HPLC. Its calculated mass was 1742.7 and observed was 1742.6. The cleavage Cocktail 4 was the most effective, and Cocktail 3 had the least yield. It seems one hour of reaction time is enough for complete cleavage with the highest yield compared with two- and three-hour reactions. Cocktails 1 and 2 showed similar efficiency (*p* < 0.01). Cleavage Cocktail 1 also gave a comparable yield in the two-hour reaction time at 1 h (*p* < 0.01). In general, longer reaction times decrease yield, which might increase side reactions. Then, Cocktail 4 and 1 h reaction was used in all the rest resin-bounded peptide cleavage, and the linear peptide was used in subsequently described studies.

**Table 1:** Four kinds of different Fmoc cleavage cocktails. TFA, trifluoroacetic acid; TIPS, triisopropylsilane; EDT, 1,2-ethanedithiol.

NO.	Recipe
Cleavage Cocktail 1	TFA/phenol/water/TIPS (264/15/15/6 μL, v⁄v⁄v⁄v)
Cleavage Cocktail 2	TFA/water/TIPS (279/15/6 μL, v⁄v⁄v)
Cleavage Cocktail 3	TFA/water (285/15 μL, v⁄v)
Cleavage Cocktail 4	TFA/phenol/water/thioanisole/EDT (247.5/15/15/7.5 μL, v⁄v⁄v⁄v/v)

#### 2.1.2. Two-step Oxidative Folding of α-CTx TxIB Linear Peptide

**Figure 3:**
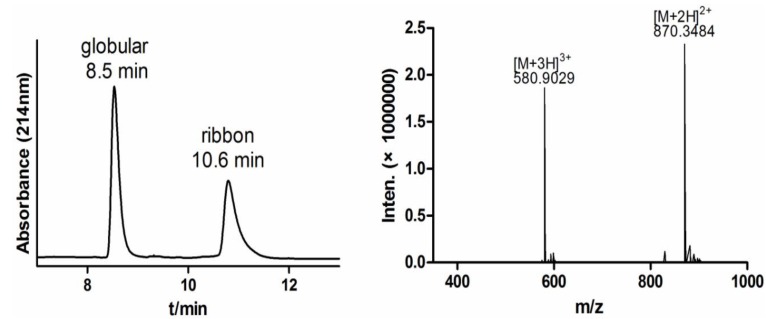
HPLC and ESI-MS mass spectrometry analysis of the TxIB “globular” isomer and “ribbon” isomer by two-step oxidative folding. (**A**) HPLC traces of globular and ribbon TxIB. Analytical HPLC was carried out using an analytical Vydac C18 (5 μm, 4.6 mm × 250 mm). The separation method was as follows: a 10%–30% solvent B gradient over 30 min (solvent B is 0.05% TFA in 90% ACN; solvent A is 0.1% TFA in H_2_O). (**B**) ESI-MS analysis of the globular and ribbon isomers. Its calculated mass was 1739.70.

α-CTx TxIB shares a conserved cysteine pattern, CC-C-C, with four cysteine residues. There are three possible disulfide bond connectivities for α-CTxs, which is referred to as the native “globular” form to distinguish it from the alternative “ribbon” or “bead” connectivities (Figure 1). The two-step oxidation folding was used to fold linear α-CTx TxIB selectively to produce “globular” and “ribbon” isomers, respectively. We protected the cysteine side chains with two orthogonal protecting groups that could be removed selectively under different conditions, allowing the formation of one disulfide bridge at a time. For this purpose, Cys1 and Cys3 for the “globular” isomer and Cys2 and Cys3 for the “ribbon” isomer were introduced as the acid labile S-trityl protected amino acids, whereas Cys2 and Cys4 for the “globular” isomer and Cys1 and Cys4 for the “ribbon” isomer were *S*-acetamidomethyl cysteine. The acid-labile groups were removed simultaneously with cleavage from the resin; ferricyanide was used to oxidize the first disulfide bond. Reverse-phase HPLC was used to purify the monocyclic peptide; subsequently, the acid-stable acetamidomethyl groups were removed from the second and fourth cysteines for the “globular” isomer and from the first and fourth cysteines for the “ribbon” isomer, by iodine oxidation, which simultaneously formed the second disulfide bond. The two fully oxidized isomers were purified by HPLC (Figure 3). The TxIB “globular” isomer is the first peak in Figure 3, with a disulfide bond connectivity linking Cys1–3, Cys2–4, and its retention time was 8.5 min. The TxIB “ribbon” isomer is the second peak in Figure 3 with a disulfide bond connectivity linking Cys1–4 and Cys2–3, and its retention time was 10.6 min. This is the first time the ribbon isomer of α-CTx TxIB has been synthesized, which would help to identify the right isomer from random one-step oxidative folding products. ESI-MS mass spectra of synthetic α-CTx TxIB “globular” isomer and “ribbon” isomer were consistent with the sequence. The two isomers of TxIB have the same mass, which was calculated as 1739.70 and observed as 1739.71 for “globular” and 1739.70 for “ribbon”.

#### 2.1.3. One-Step Oxidative Folding of α-CTx TxIB Linear Peptide

**Figure 4:**
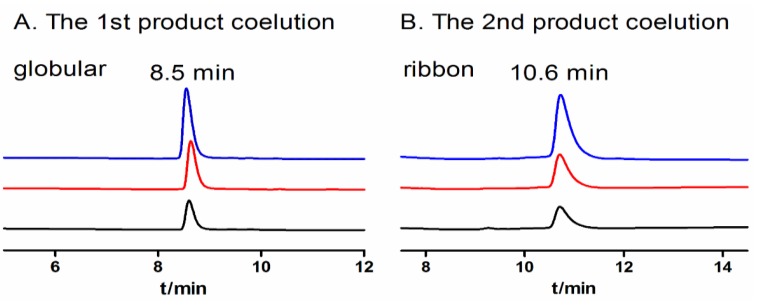
Coelution of “globular” isomer and “ribbon” isomer from one-step oxidation and two-step oxidation, respectively. (**A**) The first product of one-step oxidation and the native “globular” isomer of two-step oxidation were co-eluted in a 1:2 ratio and analyzed by HPLC. (**B**) The second product of one-step oxidation and the “ribbon” isomer of two-step oxidation were co-eluted in a 1:2 ratio and analyzed by HPLC. The retention time with 8.5 min of “globular” isomer (A) and 10.6 min of “ribbon” isomer (B) was the same for one-step oxidation (black), two-step oxidation (red) and the co-eluted peptides (blue) respectively.

When one-step air oxidation folding in regular 0.1 M Tris-HCl buffer (pH 8.7, RT) was used for TxIB folding, there were two products presented corresponding to the two isomers of the peptide (Table 2, Run 1). Each isomer was then purified by HPLC. Then, two co-elution experiments were performed with an approximately 1:2 ratio of the first and the second product of the one-step air oxidation and the “globular” and “ribbon” isomers of the two-step directed oxidation, respectively (Figure 4). As shown in Figure 4A, the first product of the one-step oxidation showed an identical elution profile with the native “globular” peptide of the two-step directed oxidation, of which the retention time was 8.5 min. The second product of the one-step and “ribbon” isomer of two-step directed oxidation had identical elution profiles with a retention time of 10.6 min (Figure 4B). Thus, the first product of the equilibrium air-oxidation folding of the peptide has the disulfide bond configuration, Cys1–3 and Cys2–4, of native “globular” α-conotoxins. The second product of the one-step oxidation of the peptide has the disulfide bond configuration, Cys1–4 and Cys2–3, of the “ribbon” isomer. There was no “bead” isomer detected with the disulfide bond arrangement, Cys1–2 and Cys3–4, among one-step oxidation products of α-CTx TxIB. The yield of the folding was calculated from one of the isomer’s percent of total folded products generated from 500 nmol linear TxIB. The native “globular” isomer’s yield with air oxidation was 66%, and the “ribbon” isomer’s yield was 7% (Table 2, Run 1).

**Table 2:** Proportion of globular and ribbon isomers of α-conotoxin TxIB under 54 oxidative conditions. The folding reactions were performed for 24 h.

Oxidation Condition						TxIB	
Run	Buffer	Redox Reagent	pH	Temp	Cosolvent/Salt	G(%)	R(%)
52	0.1 M NH_4_HCO_3_	GSH:GSSG (2:1)	8	4 °C	40% DMSO	100	0
50	0.1 M Tris-HCl	GSH	8.7	4 °C	40% DMSO	100	0
51	0.1 M NH_4_HCO_3_	GSH:GSSG (2:1)	8	4 °C	30% DMSO	96	2
49	0.1 M Tris-HCl	GSH	8.7	4 °C	30% DMSO	94	3
32	0.1 M NH_4_HCO_3_	GSH:GSSG (2:1)	8	4 °C	-	91	8
34	0.1 M NH_4_HCO_3_	GSH:GSSG (2:1)	9	4 °C	-	90	8
24	0.1 M Tris-HCl	GSH	8.7	4 °C	-	88	12
12	0.1 M NH_4_HCO_3_	GSH:GSSG (2:1)	8	RT	-	88	11
35	0.1 M NH_4_HCO_3_	GSH:GSSG (2:1)	9	RT	-	87	12
20	0.1 M Tris-HCl	GSH	7	37 °C	-	86	11
31	0.1 M NH_4_HCO_3_	GSH:GSSG (2:1)	7	37 °C	-	86	11
21	0.1 M Tris-HCl	GSH	8	4 °C	-	83	14
33	0.1 M NH_4_HCO_3_	GSH:GSSG (2:1)	8	37 °C	-	83	14
30	0.1 M NH_4_HCO_3_	GSH:GSSG (2:1)	7	RT	-	83	13
45	0.1 M NH_4_HCO_3_	GSH	8	RT	30% IPA	83	7
11	0.1 M NH_4_HCO_3_	GSH:GSSG (1:1)	8	RT	-	82	11
13	0.1 M NH_4_HCO_3_	GSH:GSSG (5:1)	8	RT	-	82	13
8	0.1 M NH_4_HCO_3_	-	8	RT	-	82	13
25	0.1 M Tris-HCl	GSH	8.7	37 °C	-	82	8
29	0.1 M NH_4_HCO_3_	GSH:GSSG (2:1)	7	4 °C	-	81	15
2	0.1 M Tris-HCl	GSH	8.7	RT	-	80	14
9	0.1 M NH_4_HCO_3_	GSH	8	RT	-	80	11
36	0.1 M NH_4_HCO_3_	GSH:GSSG (2:1)	9	37 °C	-	80	15
3	0.1 M Tris-HCl	GSSG	8.7	RT	-	79	15
4	0.1 M Tris-HCl	GSH:GSSG(1:1)	8.7	RT	-	77	16
5	0.1 M Tris-HCl	GSH:GSSG (2:1)	8.7	RT	-	77	17
39	0.1 M NH_4_HCO_3_	GSH:GSSG (2:1)	8	4 °C	2 M (NH_4_)_2_SO_4_	77	21
22	0.1 M Tris-HCl	GSH	8	RT	-	76	17
23	0.1 M Tris-HCl	GSH	8	37 °C	-	76	11
10	0.1 M NH_4_HCO_3_	GSSG	8	RT	-	75	22
14	0.1 M NH_4_HCO_3_	GSH:GSSG (10:1)	8	RT	-	75	11
19	0.1 M Tris-HCl	GSH	7	RT	-	75	14
18	0.1 M Tris-HCl	GSH	7	4 °C	-	72	13
6	0.1 M Tris-HCl	GSH:GSSG (5:1)	8.7	RT	-	70	15
54	0.1 M NH_4_HCO_3_		8	4 °C	30% DMSO	67	19
1	0.1 M Tris-HCl	-	8.7	RT	-	66	7
37	0.1 M Tris-HCl	GSH	8.7	4 °C	2 M (NH_4_)_2_SO_4_	64	25
7	0.1 M Tris-HCl	GSH:GSSG (10:1)	8.7	RT	-	62	13
48	0.1 M NH_4_HCO_3_	GSH:GSSG (2:1)	8	4 °C	50% IPA	60	23
53	0.1 M Tris-HCl		8.7	4 °C	30% DMSO	54	46
43	0.1 M Tris-HCl	GSH	8.7	4 °C	30% IPA	40	52
47	0.1 M NH_4_HCO_3_	GSH:GSSG (2:1)	8	4 °C	30% IPA	35	58
17	0.1 M Tris-HCl	GSH	6	37 °C	-	31	11
41	0.1 M Tris-HCl	GSH	8.7	RT	30% IPA	31	63
38	0.1 M Tris-HC	GSH	8.7	4 °C	2 M CaCl_2_	31	61
40	0.1 M NH_4_HCO_3_	GSH:GSSG (2:1)	8	4 °C	2 M CaCl_2_	30	62
28	0.1 M NH_4_HCO_3_	GSH:GSSG (2:1)	6	37 °C	-	23	17
16	0.1 M Tris-HCl	GSH	6	RT	-	16	5
42	0.1 M Tris-HCl	GSSG	8.7	RT	30% IPA	16	73
44	0.1 M Tris-HCl	GSH	8.7	4 °C	50% IPA	16	84
27	0.1 M NH_4_HCO_3_	GSH:GSSG (2:1)	6	RT	-	4	4
46	0.1 M NH_4_HCO_3_	GSSG	8	RT	30% IPA	4	94
15	0.1 M Tris-HCl	GSH	6	4 °C	-	ND	ND
26	0.1 M NH_4_HCO_3_	GSH:GSSG (2:1)	6	4 °C	-	ND	ND

G: globular isomer; R: ribbon isomer; ND: not determined; RT: room temperature; GSH: reduced glutathione; GSSG: oxidized glutathione; DMSO: dimethyl sulfoxide; IPA: isopropanol.

#### 2.1.4. Optimization of One-Step Oxidative Folding

To increase the yield of native “globular” α-CTx TxIB, 54 kinds of oxidative folding solution were tested on one-step oxidative folding, which involved different buffers, redox reagents, pH, temperature, co-solvents and reaction times (Table 2). Several series of experiments were conducted to find optimal conditions for TxIB one-step oxidative folding, which focused on the effect of different ratios of the redox agents, reduced glutathione (GSH) and oxidized glutathione (GSSG), the effect of co-solvents, isopropanol (IPA) and dimethyl sulfoxide (DMSO), the effect of high salt concentration, as well as the effect of reaction temperature and pH. All the results of 54 kinds of oxidative folding conditions are shown in Table 2 arranged according to “globular” yield (%) in decreasing sequence. 

In initial analysis, GSH (0.5 mM), GSSG (0.5 mM) alone and different ratios of GSH and GSSG (1:1, 2:1) were used to optimize the yield of native globular isomer, which were runs 2–5 in 0.1 M Tris-HCl buffer and runs 9–12 in 0.1 M NH_4_HCO_3_ buffer at room temperature (RT). The results showed that condition 12 (0.1 M NH_4_HCO_3_, pH 8, GSH (1 mM):GSSG (0.5 mM)) was the best run for globular isomer accumulation with 88% yield. The oxidative folding required 24 h to be completed. However, folding reactions extending for more than 24 h did not increase the globular yield. To examine whether a higher GSH-GSSG ratio might favor the folding of the native isomer, other ratios of GSH and GSSG (5:1, 10:1) were tested (runs 6–7, 13–14). None of these conditions produced improvement over condition 12.

**Figure 5:**
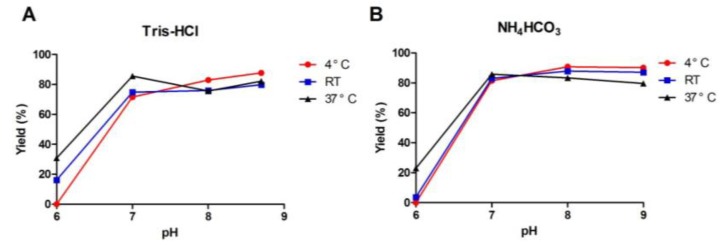
Effect of temperature and pH on globular isomer yield in Tris-HCl buffer (**A**) and in NH_4_HCO_3_ buffer (**B**). The pH clearly influenced folding yield. At higher pH, a much higher proportion of correctly folded isomer was obtained, while temperature is a minor factor in oxidative folding. The pH 8 and 4 °C were optimal for TxIB oxidative folding.

Figure 5 showed the effect of temperature and pH on folding of linear TxIB in two different buffers with redox reagents GSH (0.5 mM for Tris-HCl; 1 mM for NH_4_CO_3_) and GSSG (0.5 mM). All the experimental data showed that the buffer pH clearly influenced folding yield, but the choice of buffer was not significant on the yield. At pH 6, low or no folding yield was obtained. At pH 7–9, a much higher proportion of the correctly folded isomer “globular” was obtained. For example, in NH_4_HCO_3_ buffer (Figure 5B), no folding was observed at pH 6, and a maximum yield of 91% was obtained at pH 8. Furthermore, although in general, temperature is a minor factor in oxidative folding of α-conotoxins, we observed that temperature, in combination with pH, had a slight, but noticeable effect on TxIB folding. In Tris-HCl buffer at pH 6, no folding was observed at 4 °C; a yield of 16% was obtained at RT; and a yield of 31% was obtained at 37 °C (Figure 5A). Overall, we concluded that pH 8 and 4 °C were optimal for TxIB oxidative folding.

We examined the effect of salt on folding reaction yield by testing 2 M (NH_4_)_2_SO_4_ and 2 M CaCl_2_ (runs 37–40). For TxIB folding, (NH_4_)_2_SO_4_ addition did not significantly improve folding yield and did not change the relative yield of the globular isomer. However, CaCl_2_ changed the relative yields of globular and ribbon isomers, which increased the yield of the ribbon isomer. We also tested the effect of co-solvents, IPA and DMSO, on folding reactions. We observed that, in contrast to other studies [22], IPA addition significantly decreased the yield of globular isomer (runs 41–48). IPA addition improved the yield of ribbon isomer yield, suggesting that IPA is a key factor for oxidative folding of the ribbon isomer. In the presence of IPA and GSH or GSSH, a 63%–94% yield of ribbon isomer could be obtained (runs 41, 42, 44 and 46). Furthermore, we found that DMSO addition significantly increased the yield of globular isomer. In the presence of DMSO and GSH or GSH:GSSG, a 94%–100% yield of globular isomer was obtained (runs 49–52).

#### 2.1.5. CD Spectra of α-CTx TxIB Isomers

**Figure 6:**
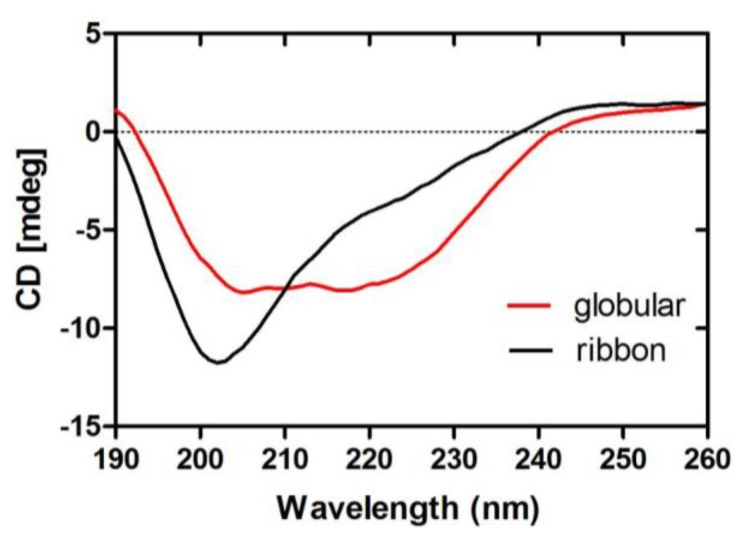
Circular Dichroism (CD) spectra of globular and ribbon isomers of α-CTx TxIB. The globular isomer (red) displayed a double minimum around at 208 and 222 nm, and the ribbon isomer (black) displayed a minimum at 202 nm.

Following peptide folding and purification, we investigated secondary structure conformation of the two TxIB isomers by circular dichroism (CD) spectra analysis. Globular isomer displays a double minimum around at 208 and 222 nm, characteristic for the α-helical structure; it also has a minimum at approximately 216 nm, characteristic of the β-sheet structure (Figure 6). In contrast, CD spectra of the ribbon isomer differed noticeably from the globular isomer. The ribbon isomer displays a minimum at 202 nm, suggesting the presence of a random coil structure; a negative maximum around 200 nm generally indicates random coil conformation [23,24]. We used the CDSSTR program to calculate secondary structures of two isomers. Compared to the globular isomer, the ribbon isomer has a lower percentage of α-helix and β-turns, while having a higher percentage of β-sheet structure (Table 3).

**Table 3:** Secondary structures of globular and ribbon isomers of α-CTx TxIB.

Isomer	Secondary structure			
	α-helix	β-sheet	β-turns	random coil
globular	15%	24%	28%	33%
ribbon	12%	29%	26%	33%

### 2.2. Discussion

**Figure 7:**
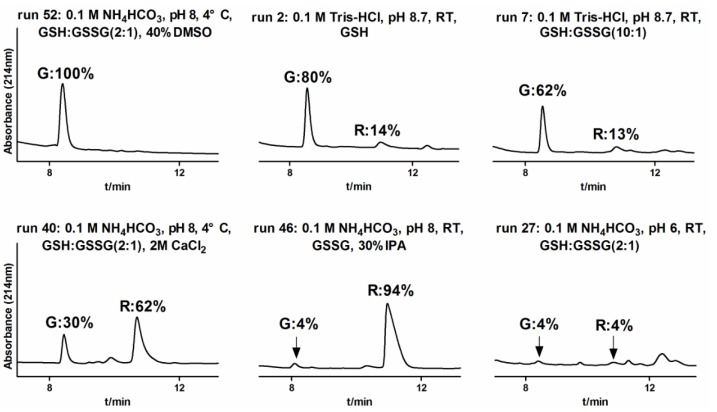
Effect of several different conditions of oxidative folding on the yield of α-CTx TxIB globular (G) and ribbon (R) isomers (runs 52, 2, 7, 40, 46, 27). The globular isomer (G) can complete oxidative folding in condition 52 with 100% yield, while there was almost no folded peptide in condition 27 with 4% yield only for each isomer. Interestingly, the ribbon isomer (R) yield reached 94% in condition 46.

α-conotoxins are small cysteine-rich peptide toxins, which have been used as important research tools for studying numerous pharmacological disorders and for designing new drugs. Such studies require large quantities of appropriately folded, bioactive peptides. Thus, developing efficient *in vitro* folding methods is a necessary first step for conducting therapeutic activity tests. In this study, we optimized cleavage conditions for α-CTx TxIB and examined key parameters influencing oxidative folding of TxIB for the first time, such as buffer, redox agents, pH, salt, co-solvent and temperature. We found that different conditions had various yields (4~100%) of oxidative folding for the globular isomer (Figure 7, Table 2). We also compared our findings with prior studies on the oxidative folding of various bioactive peptides, such as cyclotides [25,26,27] and other various conotoxins [28,29,30]. Complete oxidative folding of the α-CTx TxIB globular isomer was achieved when the NH_4_HCO_3_ or Tris-HCl folding buffers at 4 °C contained 40% of the co-solvent, DMSO, and GSH:GSSG (2:1) or GSH only with pH 8~8.7.

Initially, one-step oxidative folding of α-CTx TxIB was attempted under 0.1 M Tris-HCl, pH 8.7 (run 1) and 0.1 M NH_4_HCO_3_, pH 8 (run 8). However, we observed the accumulation of the ribbon isomer that had the same molecular mass as the native peptide, but differed significantly in retention times (Figure 3, Figure 4B). However, there was no presence of the bead isomer (Figure 3). Formation of the correct disulfide bond is extremely important in maintaining structure and activity. Generally, a disulfide bond between adjacent cysteines is energetically unfavorable, which may result in no accumulation of the bead isomer [31,32,33,34]. CD spectra showed distinct differences between the correctly folded globular isomer and the misfolded ribbon isomer of α-CTx TxIB (Figure 6). The misfolded isomer appears to be more hydrophobic, possibly due to the greater degree of exposure of hydrophobic residues compared to the compactly folded, globular isomer.

During oxidative folding of peptides, disulfide bonds are formed by multistep, reversible thiol-disulfide exchange reactions. Prior studies suggested that oxidation yield may be influenced by the GSH:GSSG ratio, which facilitates thiol-disulfide exchange during the oxidation process [35,36]. We systematically evaluated the effect of different ratios of GSH and GSSG. In these experiments, the concentration of GSH was varied, while the GSSG concentration was kept constant. The results showed that the folding rate increased by increasing the ratio of GSH and GSSG from 1:1 to 2:1, in contrast to the folding rate decreasing by increasing the ratio of GSH and GSSG to 10:1. GSH plays a key role by helping break up non-native disulfide bonds and ensuring the reversibility of the thiol-disulfide exchange. Such disulfide reshuffling is essential for the formation of the final, stably folded native product [27].

As observed by other researchers for α-conotoxin folding, temperature is not a significant factor for globular isomer yield, while the pH plays a key role [22]. Overall, our results of temperature and pH experiments are consistent with prior studies for α-conotoxins. Interestingly, at unfavorable pH conditions (pH 6), varying temperature had a slight, but noticeable effect on TxIB folding. The globular isomer is preferentially formed at lower temperature (runs 24, 32), similar to the results of Shigeru Kubo and co-workers, who have shown that ω-conotoxin MVIIC also preferentially formed the native isomer at lower temperatures [37].

pH variation, by influencing thiol-disulfide exchange reactions, frequently has a notable effect on *in vitro* peptide folding. Higher pH may facilitate efficient oxidative folding by regulating the degree of ionization of thiols to thiolates; disulfide bond reshuffling is also more significant at higher pH [38,39,40]. In this study, we concluded that pH 8 was optimal for oxidative folding of TxIB in NH_4_HCO_3_ buffer, while pH 9 also exhibited a higher proportion of globular isomer. At pH below 7, low or no folding yield was obtained. We hypothesized that the thiol-disulfide exchange reactions are effective at higher pH, while lower pH affects thiol ionization and hampers these reactions.

Optimizing redox agent concentration, pH and temperature was not sufficient to obtain 100% yield of the globular isomer, as under all these conditions, some amount of “ribbon” was always obtained. We further attempted to include salt or co-solvent to reduce the amount of ribbon product and increase the yield of globular isomer. Prior research about ω-conotoxin had demonstrated that salt reduced the repulsive forces between positive charges, which improve native isomer yield. Accordingly, the use of salts, such as (NH_4_)_2_SO_4_ and CaCl_2_, facilitates oxidative folding. In this work, neither (NH_4_)_2_SO_4_ nor CaCl_2_ in oxidative folding significantly improved folding yield of the globular isomer. Previously, Kubo *et al.* [41] had also shown that no significant improvements in refolding of HNP1 were observed by changing ammonium salts. In contrast, including CaCl_2_ led to a greater yield of the α-CTx TxIB ribbon isomer. It is likely that three positively charged residues (R9, K11, H12) and two negatively charged residues (D5, D14) of α-CTx TxIB are both on the globular isomer surface; the repulsive forces are weakened due to a large distance among the same kind of charged residues.

Co-solvent is a key factor reported in the literature for oxidative folding of bioactive peptides [25,32,38,42]. In order to shift the equilibrium towards the production of the native isomer, we tested IPA and DMSO to improve the yield of the globular isomer. All disulfide bonds in the ribbon isomer have to be reshuffled for globular isomer folding, an extensive rearrangement that must be combined with major conformational changes. Both co-solvents have a similar function in the reaction buffer by solvating hydrophobic side chains [43,44]; however, they had distinct effects on α-CTx TxIB folding. Notably, we found that IPA significantly decreased native isomer yield, while increasing ribbon isomer yield. These results are different from other α4/7-conotoxins, such as α-CTx MII, α-CTx PnIB and α-CTx GID, for which IPA significantly increased the accumulation of the globular isomer [22]. We hypothesize that IPA presence in the folding reaction may preferentially form more hydrophobic isomers of α-CTx TxIB.

In comparison, DMSO assisted the folding of the α-CTx TxIB globular isomer. Inclusion of DMSO as a co-solvent significantly increased the accumulation of the globular isomer (runs 49–52). Inclusion of 40% DMSO allowed for complete oxidative folding of the α-CTx TxIB globular isomer. These results are similar to the findings of Tam [38], whereby the use of DMSO can significantly increase native isomer yield for basic and hydrophobic disulfide-rich peptides, a result that cannot be obtained by varying redox agent ratios only. However, using DMSO alone, without GSH, is also not sufficient (runs 53–54). We hypothesize that while DMSO facilitates globular isomers, it cannot promote disulfide bond reshuffling and limits the accumulation of the ribbon isomer.

## 3. Experimental Section

### 3.1.Materials

Phenol, thioanisole, triisopropylsilane (TIPS), 1,2-ethanedithiol (EDT) and diethyl ether were from Tiandi (Haikou City, China). Reverse phase HPLC analytical Vydac C18 (5 μm, 4.6 mm × 250 mm) and preparative C18 Vydac columns (10 μm, 22 mm × 250 mm) were obtained from Shenyue (Shanghai City, China). Reagents for peptide synthesis were from GL Biochem (Shanghai, China). Acetonitrile was from Fisher (Fisher Scientific Company L.L.C., Pittsburgh, PA, USA), and trifluoroacetic acid was from Tedia (Fairfield, OH, USA).

### 3.2.Resin-Bounded Peptide Synthesis and Cleavage

The resin-bounded peptide of α-CTx TxIB was synthesized by one-step oxidation and two-step oxidation, respectively. The peptide was synthesized on an amide resin by solid-phase methodology on an ABI 433A peptide synthesizer using Fmoc (*N*-(9-fluorenyl) methoxycarbonyl) chemistry. For one-step oxidation, all cysteine residues were trityl protected. For two-step oxidation, Cys residues were protected in pairs with either *S*-trityl on Cys1 and Cys3 or S-acetamidomethyl on Cys2 and Cys4, as described previously [15]. The crude peptide was cleaved from the resin and precipitated with four different Fmoc cleavage cocktails at 3 different cleavage times, respectively (Table 1). Briefly, 25 mg of the TxIB resin-bounded peptide was placed in 10 mL tubes and was treated with four different cleavage cocktails for 1 h, 2 h and 3 h at room temperature (RT). All cleavage cocktails and scavengers were freshly prepared prior to use. The resin was removed by filtration, then the support was rinsed with TFA. Ether-peptide mixture was incubated at 4 °C overnight after the combined filtrates were added to cold (−20 °C) diethyl ether. The precipitated peptide was collected by centrifugation, which was linear TxIB peptide after being lyophilized. The linear peptide was dissolved with 2 mL HPLC-grade water and 10 μL was injected into reverse-phase HPLC (RP-HPLC) for analysis. ESI-MS mass spectrometry was utilized to confirm the identity of the products.

### 3.3. Two-Step Oxidation Folding

A two-step oxidation protocol was used to fold linear α-CTx TxIB selectively. The first disulfide bond between Cys1 and Cys3 (globular) or Cys2 and Cys3 (ribbon) was formed by adding peptide into an equal volume of 20 mM potassium ferricyanide, (K_3_[Fe(CN)_6_]), 0.1 M Tris, pH 7.5, and mixing for a 45 min reaction at room temperature. The monocyclic peptide was purified by reverse-phase HPLC (RP-HPLC). In order to remove S-Acm groups and simultaneously form the second disulfide bond, between Cys2 and Cys4 (globular) or Cys1 and Cys4 (ribbon), purified peptide was used for iodine oxidation as follows: peptide was dripped into an equal volume of 10 mM iodine in H_2_O:TFA:ACN (73:3:24 by volume) and stirred for 10 min with N_2_. The reaction was terminated by addition of ascorbic acid until colorless, and the final peptide was purified by RP-HPLC using a Vydac C18 column. The HPLC separation method was a linear gradient of 10%–30% solvent B gradient over 30 min (solvent B is 0.05% TFA in 90% ACN; solvent A is 0.1% TFA in H_2_O). ESI-MS mass spectrometry was utilized to confirm the identity of the products.

### 3.4. One-Step Oxidative Folding

A one-step oxidation protocol was used to fold linear α-CTx TxIB randomly. Various folding conditions were investigated to fold the peptide directly, including different buffers, redox reagents, GSH/GSSG, pH, temperatures, salt and co-solvents (Table 2). For each oxidation condition, final concentration of linear TxIB in each trial was 50 nM/mL. At 1-h, 2-h, 4-h, 24-h and 48-h intervals, samples were acidified with formic acid to terminate the folding process. Folding product profiles were analyzed by analytical RP-HPLC with a 10%–30% solvent B gradient over 30 min. For reference comparison, correctly folded globular isomer and other misfolded isomers were identified by co-injections of pure isomers obtained by two-step synthesis. A Shimadzu HPLC system was used to calculate the HPLC profile peak area. Folding yields were determined as a ratio of the steady state accumulation of native species, relative to all folding species.

### 3.5. Circular Dichroism (CD) Measurements

Individual isomers were purified by HPLC. CD spectra of globular and ribbon isomers were measured at the 0.35 mg/mL peptide concentration in HPLC-water (pH 7.0) using a Jasco J-810 spectropolarimeter at room temperature. Spectra were collected at 0.5 nm intervals over the wavelength range 260–190 nm in a 0.1 cm path length cuvette.

## 4. Conclusions

In summary, the current study focused on optimizing conditions for high-yield production of the correctly folded native globular isomer of α-CTx TxIB, which is a uniquely selective ligand blocking α6/α3β2β3 nAChRs. The results showed that inclusion of GSH, cosolvent, DMSO, basic pH conditions and lower temperature significantly improved the yield of the TxIB native isomer. The most optimized folding conditions were obtained for one-step oxidation synthesis of α-CTx TxIB, which produced the native globular isomer only and excluded the other by-products of misfolded isomers. These findings would serve as a foundation to synthesize α-CTx TxIB efficiently at a large scale for future testing of potential therapeutic properties and its biomedical applications.

## Abbreviations

DMSO: dimethyl sulfoxide
GSH: reduced glutathione
GSSG: oxidized glutathione
HPLC: high performance liquid chromatography
ESI-MS: electrospray ionization mass spectrum
ACN: acetonitrile
CDSSTR: a program for estimating protein secondary structure fractions
α-CTx: α-conotoxin
